# Derangement of Liver Enzymes, Hyperglycemia, Anemia, and Associated Factors among HIV-Infected Patients Treated with Tenofovir Disoproxil Fumarate-Based Regimen in Ethiopia: A Prospective Cohort Study

**DOI:** 10.1155/2021/6613519

**Published:** 2021-06-16

**Authors:** Taklo Simeneh Yazie

**Affiliations:** Pharmacology Unit and Research Team, Department of Pharmacy, College of Health Sciences, Debre Tabor University, P.O. Box 272, Debre Tabor, Amhara, Ethiopia

## Abstract

Hepatotoxicity was found in different case reports and studies in tenofovir disoproxil fumarate- (TDF-) based regimen. However, there was no data regarding liver enzymes, glucose, and hemoglobin in Ethiopian patients receiving TDF-based regimen. The aim of this study was to determine elevated liver enzymes and its associated factors as well as elevated fasting plasma glucose and anemia. A hospital-based observational prospective cohort study was conducted on conveniently selected 63 patients in Tikur Anbessa Specialized Hospital (TASH) from January to September 2019. Laboratory values were determined at pre-TDF-based regimen baseline and six-month follow-up. The data was analyzed by using SPSS version 21.0, and multivariate logistic regression was used to determine associated factors with elevated liver enzymes. The overall elevated liver enzymes were found in 26 (41.3%) participants. From this, elevated alanine aminotransferase (ALT), aspartate aminotransferase (AST), and alkaline phosphatase (ALP) comprise 3 (4.8%), 3 (4.8%), and 20 (31.8%), respectively. Elevated fasting plasma glucose (FPG) was found in 9 (14.3%) and 14 (22.2%) of participants at baseline and six-month visit, respectively. At six-month visit, 4 (6.4%) of participants experienced anemia. The mean value of ALP and FPG at six months was significantly higher than their respective baseline mean values (mean difference (MD) = +63.38, 95% CI (39.84, 86.92), *p* = 0.0001; MD = +6.64, 95% CI (2.63, 10.64), *p* = 0.002, respectively). The mean value of ALT, AST, and Hg at six months was slightly increased compared to their respective baseline mean values, but the difference was not significant. In multivariate analysis, only female sex was significantly associated with elevated ALP (AOR = 4.5, 95% CI (1.03, 19.6), *p* = 0.045). Overall mild and moderate hepatotoxicity was found to be high (26, 41.3%) in the present study, and from this, the majority was comprised by elevated ALP (20, 31.8%). The proportion of participants with hyperglycemia was increased at the end of follow-up compared to its baseline value, but anemia was not. Female sex was significantly associated with elevated ALP. This study warrants monitoring of liver enzymes and glucose in TDF-based regimen.

## 1. Introduction

Drugs are significant cause of liver injury. Around 75% of the idiosyncratic drug reactions result in liver transplantation or death. Different types of drug induced liver diseases are acute dose dependent liver damage, acute fatty infiltration, cholestatic jaundice, liver granulomas, active chronic hepatitis, liver cirrhosis, and liver tumors. In the United States, nearly 2000 cases of acute liver failure occur annually, and drugs account for over 50% of them [1]. Abnormalities of liver enzymes are found both in human immunodeficiency virus (HIV) and antiretroviral therapy (ART) [2–6]. In addition, anemia and hyperglycemia were depicted in HIV and ART [7–14].

Particularly, in tenofovir disoproxil fumarate-based regimen hepatotoxicity was revealed in case reports [15–17]. Furthermore, various studies found elevation of liver enzymes in TDF-based regimen [18–21], and a study in China found that 48.1% of participants had hepatotoxicity following TDF-based regimen [22]. Hepatic histological damage is also found in animal study [23]. Several associated factors with hepatotoxicity are identified in various studies such as concomitant use of antituberculosis drugs and ART, low CD4 count, female gender, and age over 40 years [4, 18]. In another study, male sex was associated with elevated alanine aminotransferase (ALT) and aspartate aminotransferase (AST) [5]. Currently in Ethiopia, TDF-based regimen is the preferred first line therapy, and more patients are exposed to it. However, there is no data regarding liver enzymes, glucose, and hemoglobin profiles. In addition, studies recommend liver enzyme monitoring but in Ethiopia, patients receiving ART without routine monitoring of laboratory values [18, 24]. Therefore, the present study is aimed at determining liver enzymes, glucose, hemoglobin profiles and associated factors with abnormal laboratory values in TDF-based regimen among HIV-infected patients in Tikur Anbessa Specialized Hospital (TASH).

## 2. Methods

### 2.1. Study Participants

Adult HIV-infected patients who are going to initiate TDF-based regimen and/or whose regimens are going to be switched to TDF-based regimen are enrolled consecutively for two months and being followed for six months after enrollment. The study area was TASH, and it was selected purposively because there were relatively greater number of patients initiating TDF-based regimen in TASH compared to other hospitals in Addis Ababa; the detail of the method was explained in my previous work [25]. This study was conducted from January 15 to September 15, 2019, and a total of 66 participants were enrolled, but 3 participants lost the study after baseline visit. Patients who are critically ill, pregnant, taking TDF-based regimen previously, age less than 18 years old, and cognitively impaired are excluded from the study. Written informed consent was obtained from each participant. Ethical approval was obtained from Addis Ababa University, School of Pharmacy Ethical Review Board (Ref. No. ERB/SOP/07/09/2019), and this study is conducted in accordance with Declaration of Helsinki.

### 2.2. Data Collection Procedure

A structured questionnaire was adapted from related literatures to collect socio-demographic and clinical data. First, it was written in English and then translated to Amharic. The questionnaire was retranslated to English by an expert to test its consistency. Two senior nurses and laboratory technologists took a half-day training regarding objective, methodology, and ethical issue of the study. The nurses collected data by using structured questionnaire and checklist whereas laboratory technologists determined the level of liver enzymes, glucose, and hemoglobin. Previous ART and baseline CD4 count of patients who had prior ART exposure was recorded from patient chart by using checklist. For treatment naïve patients' baseline, CD4 count was done in routine care, and it was recorded in the checklist. Weight and height were measured by using Seca 761 weight scales and height ruler (with meter reading) which is attached with it, respectively (made in Germany). Body mass index (BMI) of participants was calculated as follows: Body mass index = Weight (in kg) ÷ (Height (in m))^2^.

Liver enzymes were measured at baseline and 24 weeks according to previous study [20]. ALT, AST, alkaline phosphatase (ALP), fasting plasma glucose (FPG), and hemoglobin (Hg) were determined by a Hitachi Automatic Analyzer 7600 (Hitachi, Ltd., Tokyo, Japan) at TASH, clinical chemistry laboratory. All laboratory values were graded according to the U.S. National Institutes of Health Division of AIDS (DAIDS) grading table (version 2.1, July 2017), which grades abnormalities from 1 (mild) to 4 (potentially life-threatening). For patients with pretreatment serum ALT, AST, and ALP values within normal range, elevation of liver enzymes was classified according to changes relative to the upper limit normal value (ULN): grade 1 (1.25 to <2.5ULN), grade 2 (2.5 to <5ULN), grade 3 (5 to <10ULN), and grade 4 (≥10ULN) [26]. For patients with pretreatment elevated liver enzymes, elevation of liver enzymes was classified according to changes relative to baseline values rather than ULN: grade 1 (1.25 to 2.5 × baseline), grade 2 (2.6 to 3.5 × baseline), grade 3 (3.6 to 5 × baseline), and grade 4 (>5 × baseline) [27].

FPG values were classified as grade 1 (110 to 125 mg/dL), grade 2 (>125 to 250 mg/dL), grade 3 (>250 to 500 mg/dL), and grade 4 (>500 mg/dL) whereas Hg values were classified as grade 1 (10 to 10.9 mg/dL), grade 2 (9 to <10 mg/dL), grade 3 (7 to <9 mg/dL), and grade 4 (<7 mg/dL) for male and grade 1 (9.5 to 10.4 mg/dL), grade 2 (8.5 to <9.5 mg/dL), grade 3 (6.5 to <8.5 mg/dL), and grade 4 (<6.5 mg/dL) for female [26].

### 2.3. Statistical Analysis

The collected data was entered in to SPSS version 21, and descriptive statistics like frequency, percentage, median, and mean with standard deviation were used to describe variables. Paired *t*-test and McNemar's test were used to compare mean laboratory values and proportion of elevated laboratory values at six-month visit from baseline, respectively. Binary logistic regression was used to identify factors associated with elevated liver enzymes, and hyperglycemia. Factors with *p* value of < 0.25 in univariate binary logistic regression analysis were incorporated into multivariate binary logistic regression analysis, and statistical significance was set at *p* < 0.05.

## 3. Results

### 3.1. Study Participant Characteristics

Sixty-three study participants were included in the analysis, and among them, 43 (68.3%) were female. The majority of study participants (59, 92.1%) was treatment naïve at enrollment whereas 5 (79%) were exposed to zidovudine-based regimen, and their previous regimen is going to be switched to TDF-based regimen due to adverse effects. The mean (±standard deviation (SD)) age of the study participants was 39.7 (±10), and the median (interquartile range (IQR)) of baseline CD4 count was 241 (106-457). Regarding to comorbidity, hypertension, type 2 diabetes mellitus, cancer, and kidney stone were found in 6.3%, 3.2%, 12.7%, and 3.2%, respectively, whereas opportunistic infections were found in 19.05% of participants (Table 1).

### 3.2. Elevated Laboratory Values among Study Participants

The overall elevated liver enzymes were found in 26 (41.3%) participants. From this, elevated ALT, AST, and ALP comprise 3 (4.8%), 3 (4.8%), and 20 (31.8%), respectively. Among participants with elevated liver enzymes, female accounts 22 (34.9%). Elevated FPG was found in 9 (14.3%) and 14 (22.2%) of participants at baseline and six-month visit, respectively (but there is no significant difference between baseline and six-month prevalence, McNemar's test of *p* = 0.18). Concerning to anemia, 4 (6.4%) of participants experienced anemia at six-month visit (Table 2).

The mean value of ALP and FPG at six-month follow-up was significantly higher than their respective baseline mean values ((MD) = +63.38, 95% CI (39.84, 86.92), *p* = 0.0001; MD = +6.64, 95% CI (2.63, 10.64), *p* = 0.002, respectively). The mean value of ALT, AST, and Hg at six months was slightly increased compared to their respective baseline mean values, but the difference was not statistically significant.

### 3.3. Factors Associated with Elevated Liver Enzymes

Among factors which were included in univariate binary logistic regression analysis, BMI, CD4 count, age, and isoniazid prophylaxis fulfilled the criteria to be incorporated in multivariate analysis for elevated ALT and AST whereas for elevated ALP, CD4 count, age, sex, and isoniazid prophylaxis fulfilled the criteria to be included in multivariate analysis (Table 3).

Concerning to elevated FPG, marital status, occupation, hypertension, tuberculosis, dyslipidemia and glomerular filtration rate were entered into multivariate logistic regression based on the set criteria of *p* < 0.25. However, none of the above variables were significantly associated with elevated FPG (Table 4).

In multivariate analysis, none of the variables were significantly associated with elevated ALT, AST, and ALP except female sex which was significantly associated with elevated ALP (Table 5).

## 4. Discussion

TDF-based regimen is frequently used for the treatment of HIV/AIDS, and different studies showed abnormal values of liver enzymes, hemoglobin, and fasting plasma glucose in TDF-based regimen. In Ethiopia, particularly at the study area, there is no previous study that determines these laboratory parameters, so the present study is the first study which determines liver enzymes, hemoglobin, and fasting plasma glucose in TDF-based regimen. In the present study, hepatotoxicity was found in 26 (41.3%) study participants, and from this, grades 1 and 2 hepatotoxicity comprised of 38.1% and 3.2%, respectively. Grades 3 and 4 hepatotoxicity was not found in this study. Regarding to elevated ALT at six-month follow-up, it was found in 3 (4.8%) of participants which is lower than the results of the studies done in Namibia, India, and China [18–20, 22]. The discrepancy of the findings may be due to difference in baseline prevalence of elevated ALT, definition of elevated ALT, baseline socio-demographic, and clinical characteristics of study participants. Similar to elevated ALT, elevated AST at six months was found in 3 (4.8%) of the participants which is lower than the finding of the study done in China [22]. The difference in baseline socio-demographic and clinical characteristics, duration of follow-up, and difference in definition of elevated AST may be the reason for the disagreement of the results.

Concerning to mean value of ALT, there was slight increase at six-month follow-up compared to baseline mean value, but the difference was not significant (mean difference = +3.1, *p* = 0.148). This result is in line with the findings of the study conducted in Namibia [18, 19]. In contrast, the result of the present study is different from the result of the study done in India [20]. The disagreement of these findings may be due to difference in baseline socio-demographic and clinical characteristics of study participants.

Anemia was found in 4 (6.4%) of study participants which is lower than the findings of the studies in TASH, Debre Tabor General Hospital, and Wolaita Sodo University Teaching Referral Hospital. This disagreement may be explained in part by the difference in definition of anemia and type of ART regimens between the studies [8–10]. Regarding to the mean of Hg at six months, it was slightly increased (mean difference = +0.24, *p* = 0.326) compared to baseline mean value, but the difference was no significant. This finding is in contrast to the finding of the study done in TASH [8], and the discrepancy of these findings may be due to the difference in type of ART regimens.

Hyperglycemia was detected in 14 (22.3%) of the participants in the present study, which is in line with the result of the study done by García-benayas et al. [11]. This result was higher than the result of the study done by Erlandson et al. [12]. The difference may be in part due to difference in socio-demographic characteristics and duration of follow-up of participants. There was significant mean change (mean difference = 6.64, *p* = 0.002) in FPG at six-month follow-up compared to baseline mean values in the present study, which is in line with the finding of the study done by Erlandson et al. [12].

Among independent variables which were included in multivariate analysis, only female sex was significantly associated with elevated ALP (AOR = 4.5, 95% CI (1.03, 19.6), *p* = 0.045). This finding is similar with the finding of the study done in Namibia [18].

Mild and moderate hepatotoxicity was found in 41.3% of the study participants, and the only independent variable that was significantly associated with ALP was female sex. Significant mean change was found for ALP and FPG at the end of the follow-up compared to their respective baseline mean values. Some unknown factors may affect the outcome of the study, and the small sample size of the current study may limit its generalizability to the target population. In the future, longitudinal study with large sample size is recommended to identify more associated factors with elevated liver enzymes and to determine the prevalence of hepatotoxicity in TDF-based regimen.

## 5. Conclusion

Overall mild and moderate hepatotoxicity was found to be high (26, 41.3%) in the present study, and from this, the majority was comprised by elevated ALP (20, 31.8%). Hyperglycemia and anemia were found in 14 (22.3%) and 4 (6.4%) of participants at six-month visit, respectively. The proportion of participants with hyperglycemia was increased at the end of follow-up compared to its baseline value, but anemia was not. Female sex was significantly associated with elevated ALP. This study warrants monitoring of liver enzymes and glucose in TDF-based regimen.

## Figures and Tables

**Table 1:** Characteristics of study participants.

Variable	Category	Frequency
Age in years	>40	21 (33.3)
Body mass index (in kg/m^2^)	≥25	20 (31.75)
Baseline CD4 count	≥200 cells/mm^3^	34 (53.97)
WHO stage	I + II	45 (71.43)
	III + IV	18 (28.57)
Marital status	Single	8 (12.7)
	Married	40 (63.5)
	Divorced	11 (17.5)
	Widowed	4 (6.4)
Educational status	Illiterate	5 (7.9)
	Primary	18 (28.6)
	Secondary	28 (44.4)
	Diploma	5 (7.9)
	Degree	7 (11.1)
Occupation	Governmental employee	12 (19)
	Private	34 (54)
	Not employed	17 (27)
Cotrimoxazole px	Yes	42 (66.7)
Isoniazid px	Yes	8 (12.7)
Chemotherapy	Yes	4 (6.3)
NSAIDs	Yes	6 (9.5)
PPI	Yes	9 (14.3)
Antituberculosis drugs	Yes	4 (6.3)
Cancer	Yes	8 (12.7)

CD4: cluster of differentiation; WHO: World Health Organization; Px: prophylaxis; NSAIDs: nonsteroidal anti-inflammatory drugs; PPI: proton pump inhibitor.

**Table 2:** Elevation of laboratory values at six-month visit among study participants.

Variable	Grade of toxicity	Frequency (%)
ALT	Grade 1	2 (3.2)
	Grade 2	1 (1.6)

AST	Grade 1	3 (4.8)

ALP	Grade 1	19 (30.2)
	Grade 2	1 (1.6)

FPG	Grade 1	11 (17.5)
	Grade 2	2 (3.2)
	Grade 3	1 (1.6)

Hg	Grade 1	3 (4.8)
	Grade 2	0 (0)
	Grade 3	1 (1.6)

ALT: alanine aminotransaminase; AST: aspartate aminotransaminase; ALP: alkaline phosphatase; FPG: fasting plasma glucose; Hg: hemoglobin.

**Table 3:** Univariate binary logistic regression analysis of variables associated with elevated liver enzymes.

Variables	Category	Elevated liver enzymes		
		ALT COR (95% CI)	AST COR (95% CI)	ALP COR (95% CI)
Sex	Female	0.93 (0.08, 10.86)	NA	3.71 (0.94, 14.6)^∗^
Age	>40 years	1 (0.09, 11.7)^∗^	4.32 (0.37, 50.58)^∗^	2.11 (0.7, 6.38)^∗^
BMI	>25 kg/m^2^	4.32 (0.37, 50.58)^∗^	4.32 (0.37, 50.58)^∗^	1.54 (0.51, 4.65)
CD4	<200 cells/mm^3^	0.57 (0.05, 6.65)^∗^	0.57 (0.05, 6.65)^∗^	2.29 (0.78, 6.78)^∗^
Cart	TDF+3TC + ATV/r	NA	NA	0.84 (0.15, 4.78)
Cotrimoxazole	Yes	NA	1 (0.09, 11.7)	0.9 (0.29, 2.74)
Isoniazid	Yes	3.79 (0.3, 47.36)^∗^	3.79 (0.3, 47.36)^∗^	2.44 (0.54, 10.96)^∗^
PPI	Yes	3.25 (0.26, 40.12)	3.25 (0.26, 40.12)	1.09 (0.24, 4.88)
Prior ART	Yes	NA	NA	1.48 (0.23, 9.65)
OPIs	Yes	NA	NA	1.34 (0.29, 6.27)
Diabetes	Yes	NA	NA	2.21 (0.13, 37.25)
Hypertension	Yes	NA	NA	0.72 (0.07, 7.2)
Cancer	Yes	NA	NA	1.34 (0.29, 6.27)

BMI: body mass index; CD4: cluster of differentiation; cART: combination of antiretroviral therapy; PPI: proton pump inhibitor; OPIs: opportunistic infections; NA: not applicable. ^∗^ stands for variables with *p* value of < 0.25.

**Table 4:** Univariate and multivariate logistic regression of factors with elevated fasting plasma glucose.

Variables	Category of variables	Elevated FPG		Univariate COR, 95% CI	Multivariate AOR, 95% CI
		Yes (%)	No (%)		
Marital status	Single	3 (37.5)	5 (62.5)	1	1
	Married	6 (15)	34 (85)	0.29, 0.06-1.57^∗^	0.15, 0.01-1.88
	Divorced	3 (27.3)	8 (72.7)	0.63, 0.09-4.40	0.23, 0.02-3.29
	Widowed	2 (50)	2 (50)	1.67, 0.15-18.87	1.23, 0.05-28.71

Occupation	Government	1 (8.3)	11 (91.7)	1	1
	Private	8 (23.5)	26 (76.5)	3.39, 0.38-30.40	5.775, .153-217.275
	No work	5 (29.4)	12 (70.6)	4.58, 0.46-45.61^∗^	5.912, .166-210.497

Hypertension	Yes	1 (25)	3 (75)	13.09, 1.24-138.11^∗^	8.779, .630-122.412
	No	13 (22.4)	45 (77.6)	1	1

Tuberculosis	Yes	2 (50)	2 (50)	3.92, 0.50-30.73^∗^	1.14, 0.07-18.55
	No	12 (20.3)	47 (79.7)	1	1

Dyslipidemia	Yes	12 (26.1)	34 (73.9)	1.38, 0.08-1.90^∗^	0.54, 0.09-3.26
	No	2 (11.8)	15 (88.2)	1	1

GFR	≤90 mL/min	12 (30.8)	27 (69.2)	4.89, 0.99-24.20^∗^	5.06, 0.64-39.99
	>90 mL/min	2 (8.3)	22 (91.7)	1	1

AOR: adjusted odds ratio; COR: crude odds ratio; FPG: fasting plasma glucose; GFR: glomerular filtration rate. Note: independent variables are baseline variables. ^∗^ indicates variables fulfilled the set criteria to be included in multivariate analysis (*p* < 0.25); there is no significant association in multivariate analysis.

**Table 5:** Multivariate binary logistic regression analysis for variables associated with elevated liver enzymes.

Variables	Category	Elevated liver enzymes		
		ALT AOR (95% CI)	AST AOR (95% CI)	ALP AOR (95% CI)
Sex	Female	NA	NA	4.5 (1.03, 19.6)^∗∗^
Age	>40 years	0.46 (0.02, 9.84)	3.49 (0.22, 54.93)	1.71 (0.51, 5.71)
BMI	>25 kg/m^2^	5.51 (0.26, 116.19)	2.25 (0.14, 36.91)	NA
CD4	<200 cells/mm^3^	1.39 (0.07, 28.19)	0.64 (0.04, 9.76)	3.35 (0.97, 11.61)
Isoniazid	Yes	3.49 (0.24, 50.22)	2.67 (0.18, 40.02)	2.62 (0.48, 14.13)

BMI: body mass index; CD4: cluster of differentiation 4; NA: not applicable. ^∗∗^ stands for *p* value < 0.05.

## Data Availability

Data analyzed and used for this study are available from the author upon justifiable request.

## Abbreviations

AIDS: Acquired immunodeficiency syndrome
AOR: Adjusted odds ratio
ALT: Alanine aminotransferase
ALP: Alkaline phosphatase
ART: Antiretroviral therapy
AST: Aspartate aminotransferase
COR: Crude odds ratio
FPG: Fasting plasma glucose
TDF: Tenofovir disoproxil fumarate
TASH: Tikur Anbessa Specialized Hospital.
